# Information overload in group communication: from conversation to cacophony in the Twitch chat

**DOI:** 10.1098/rsos.191412

**Published:** 2019-10-09

**Authors:** Azadeh Nematzadeh, Giovanni Luca Ciampaglia, Yong-Yeol Ahn, Alessandro Flammini

**Affiliations:** 1 School of Informatics, Computing, and Engineering, Indiana University Bloomington, Bloomington, IN, USA; 2 Network Science Institute, Indiana University Bloomington, Bloomington, IN, USA

**Keywords:** information overload, computational social science, cognitive load, Twitch

## Abstract

As social media replace traditional communication channels, we are often exposed to too much information to process. The presence of too many participants, for example, can turn online public spaces into noisy, overcrowded fora where no meaningful conversation can be held. Here, we analyse a large dataset of public chat logs from Twitch, a popular video-streaming platform, in order to examine how information overload affects online group communication. We measure structural and textual features of conversations such as user output, interaction and information content per message across a wide range of information loads. Our analysis reveals the existence of a transition from a conversational state to a cacophony—a state with lower *per capita* participation, more repetition and less information per message. This study provides a quantitative basis for further studies of the social effects of information overload, and may guide the design of more resilient online conversation systems.

## 1. Introduction

Social media enable people to access a virtually unlimited quantity of information from a virtually unlimited set of sources. This often results in our continuous exposure to a barrage of text messages, videos and sound. The information-processing capacity of humans is, however, limited. The size of the working memory, for example, corresponds to approximately seven discrete items [1]. Likewise, physical constraints in the visual and cognitive system limit our processing speed of written texts [2,3]. The effect of cognitive limits on information processing is certainly not a recent discovery but, with the recent rise of digital communication technologies, its broader implications on interpersonal communication are less well understood.

*Information overload* can be defined as the state in which one cannot make sense or act upon additional stimuli. Scholars from different fields—neuroscience, cognitive science, information science, management science and computer science—have looked at the issue of information overload from different perspectives. Despite its considerable breadth, the literature on information overload usually focuses on the *individual* level, and often takes the perspective of the *consumption* of information. Our work brings two important elements of novelty to this literature: a group-level and production-focused analysis.

Although a large literature on the effect of information overload at the individual level exists [4–9], group-level analyses are rare. It is known for example that the number of interpersonal ties an individual can manage, both offline [10] and online [11,12], is limited, suggesting the existence of active mechanisms to cope with overload at the individual level. At the group level, however, our understanding of information overload is more sparse.

Recent studies on social contagion and e-mail communication have mainly explored information overload from the perspective of information consumption and, to some extent, information production, for example looking at the rate of replies to e-mails and tweets [13–15]. However, there are still many open questions related to information production. For example, both the structure and the textual contents of a conversation may be affected by a higher information load, potentially degrading the overall production of new information.

In this work, we estimate the effects of overload on information production in the groups conversations that take place on a large social media platform.

Social media are an excellent setting to understand how information overload affects collective consumption and production of information. Social media offer an environment for *virtual* public discussion [16], with the key difference—with respect to offline world—that a potentially limitless number of people can join a single online conversation. This new scale of mass interaction may result in levels of information overload in individuals never explored before [4,14,17].

Overload in computer-mediated communication has been previously studied in legacy media such as newsgroups [18] and Internet Relay Chat (IRC) systems [19]. As the number of messages increases, chat participants cannot consume any more information, and their chances of writing new messages decreases—for example, because replying to earlier messages becomes harder. Thus, even though the total number of messages increases, the number of messages per user should actually decrease. Assuming that users tend to avoid this kind of situation by leaving the conversation, it was predicted that the overall group size would reach an equilibrium [18,19].

Empirical observations are consistent with these predictions. Indeed, it has been found that IRC rooms are typically functional with crowds of up to 300 participants, with a maximum of about 40 of them actively talking [19]. Similar observations hold for Usenet [18], and recent ethnographic studies are in support of these conclusions too [20].

Some evidence also suggests that information overload can reshape the structure and dynamics of the discourse [21]. For example, overloaded individuals may only reply to certain topics, or reply in a shorter and simpler manner [18,22,23].

Even though previous studies show evidence that information overload has macroscopic effects on group communication, it is not clear how the structure and content of these conversations changes, especially over the full spectrum of information loads achievable by modern communication platforms. Unfortunately, the systems studied in the literature on overload do not generally attain such high data loads; samples from IRC or Usenet do not generally reach the scale required to draw strong statistical claims. Overcoming these limitations, here we analyse a large dataset of chat logs from Twitch (http://www.twitch.tv/), a popular video sharing and streaming platform (see *Methods* for more information about data).

### 1.1. Live streaming and information overload

**Figure 1.**
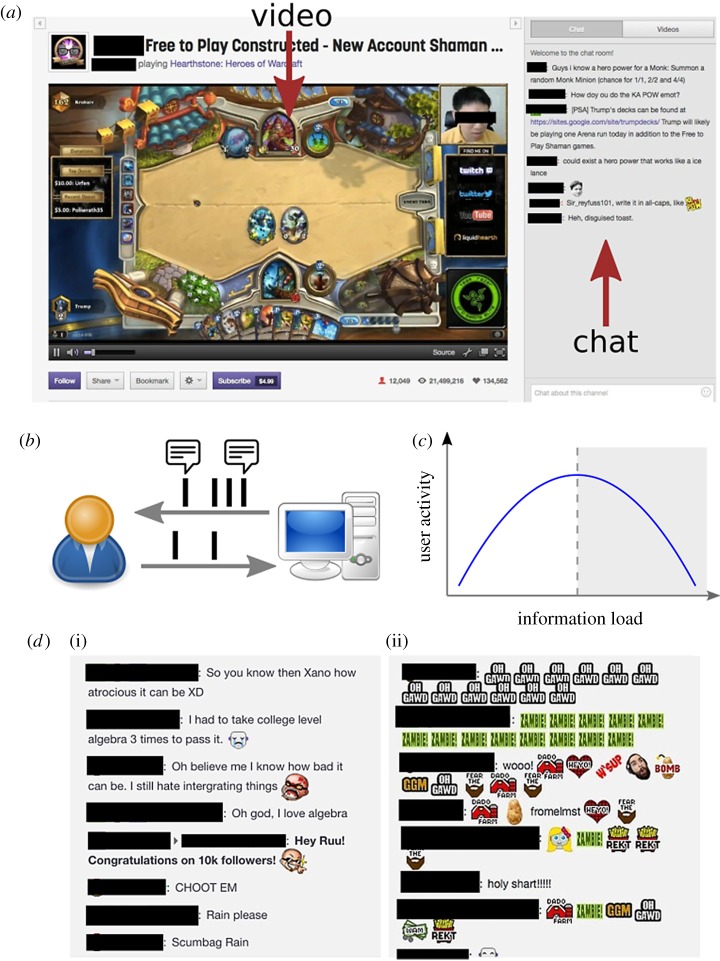
Study settings. (*a*) User interface of a Twitch stream. (*b*) Schematic illustration of the information overload in a live-streaming platform. Chat messages are information stimuli presented to the user at a certain rate (information load). A user participates in the conversation by writing messages into the chat at a certain rate (user activity). (*c*) Signature of information overload: user activity follows an inverted *U*-shape curve as a function of information load. This identifies two regimes, namely ‘conversational’ and ‘overloaded’ (shaded area). (*d*) Two excerpts of chat logs: (i) users chatting and (ii) a situation with high repetition and disproportionate usage of emotes (ii). In the overloaded regime messages will resemble more the latter than the former case.

On Twitch, people can broadcast a *stream*—usually a video feed of their screen—to other users, and share videos of past broadcasts (figure 1*a*). These users are informally called *streamers*. Both the audience and the streamer can write messages into an *interactive and real-time* chat room displayed prominently on the side of the stream. As a result, users are exposed to a live flow of messages, as in a traditional IRC channel.

Motivated by the prior research discussed above, our research question is twofold. First, the flow of messages exerts on chat participants an *information load* that needs to be processed to take an active part in the conversation (figure 1*b*). At low information loads, an increase in the load will correspond to an increase in individual *user activity*. For example, more incoming messages will elicit more replies. We call this the *conversation* regime. In the conversation regime, as the frequency of messages in the stream increases (higher load), so will the frequency of messages per user (higher activity). If the frequency of messages keeps increasing, participants cannot handle the increased information load indefinitely, and so we expect that, past a certain threshold, an increase in information load will correspond to a decrease in user activity. We call this the *overload* regime. Therefore, according to our theory, we expect to see that user activity, in the form of the frequency of messages per user, will follow an inverted *U*-shape as a function of the information load, as shown in figure 1*c*. In other words our theory predicts the existence of a threshold in the information load, past which the positive trend of user activity is inverted and becomes negative.

The second part of our research question is that the written language of the conversations will reflect the information load experienced by participants. In the conversation regime, load is manageable and it will allow participants to sustain actual conversations with replies and discourse markers (figure 1*d*). Following the flow of messages becomes harder as the information load increases, and users will resort to short cuts such as more simplified and stereotyped expressions, repetition, copy-pasting and non-textual markers, like emoticons.

This twofold question should hold for any situation in which a flow of stimuli creates an information load that needs to be processed in order to perform some kind of activity (in this case, produce additional messages). To the extent that large enough loads could be produced, one could in principle use data from any communication system to test this question. Why then is a platform like Twitch suited to do so as opposed to, say, traditional group communication systems like IRC or E-mail?

**Figure 2.**
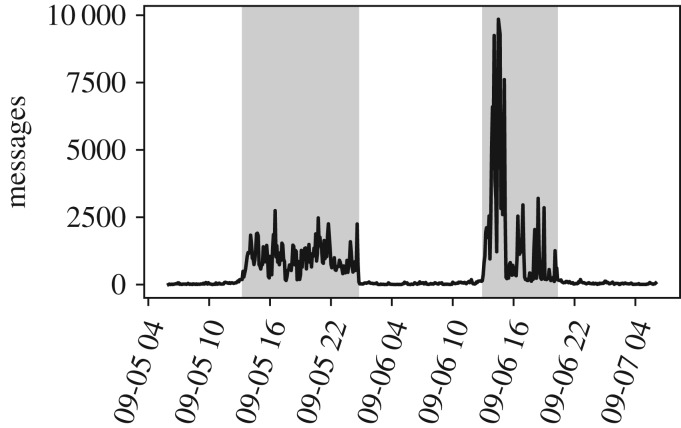
A typical time series of the volume of chat messages in a stream. We sampled the number of messages posted in the stream every 5 min. Here, peak activity corresponds to roughly 32.8 messages s^−1^. We infer the periods when a live broadcast took place (shaded areas) from sudden shifts in activity (see *Methods* for details about broadcast detection).

Unlike those systems, popular Twitch streams are often watched by massive audiences ranging in the hundreds of thousands of viewers [24], resulting in unprecedented rates of message production. In figure 2, for example, we show the rate at which messages are posted in the chat window of a stream in our dataset, sampled with a 5 min frequency, over a period of a few days. In the shaded areas, which roughly correspond to the time of live broadcasts, the peak activity reaches approximately 9845 messages (approx. 32.8 messages s^−1^).

Of course, chat activity rates do vary dramatically on Twitch, both within individual streams, and among different streams. Like in other social media platforms, the large majority of streams are watched by only a few users, while a few streams attract a huge number of viewers.^1^ In these popular streams, chat activity typically spans several orders of magnitude, depending on the time of the day and on whether there is a live broadcast or not.

In summary, using Twitch data we can examine user behaviour across a wide range of conditions—from times when messages are posted very slowly and by a handful of people, to times when a huge volume of messages is pounding the chat window. To be noted: streamers typically stream live only for a few hours a day, and for the rest of the time the stream is inactive. But, users can still log into the chat while the streamer is offline. Of course, video is an extra source of information which may cause an additional information load. Our observations could thus be affected by this. We take advantage of the fact that chats may occur also during periods of video inactivity to make sure that our results are robust against the presence of video in the stream.

We collected chat log dumps from Twitch for several days in 2014 (see *Methods* for more information about data). We computed the number of messages posted in the chat of any given stream, and the number of users who produced them, at any given time within our data window. Regarding the first part of our research question, we take the *number of messages* in the chat as a proxy for the information load, and the *messages per capita* as a proxy for user activity. For the second part, we consider a number of *metrics* of the written language of the chat (see *Methods*). Armed with these measures, we can formalize our research question into the following four operational hypotheses:

h1. Can we identify qualitatively different regimes in the way Twitch chat conversations unfold?
h2. Does a high number of messages correspond to decreased messages *per capita*, and to decreased information content, i.e. the number of bits needed to encode a message?
h3. Does the language of the chat display visible changes when the number of messages in the chat exceeds the overload threshold?
h4. Does the transition from the conversation regime to the overload regime as a function of the information load happen abruptly (i.e. discontinuous transition), or is there a more gradual deterioration?

## 2. Results

To measure information load and user activity, we aggregate our data into chunks of Δ*t* = 5 min. We define *V*_*c*_(*t*) and *U*_*c*_(*t*) (*t* = *n*Δ*t*, *n* = 0, 1, 2, … ) as the number of messages posted and the number of users who posted messages in the interval (*t*, *t* + Δ*t*), respectively. We finally define *M*(*V*) = 〈*V*_*c*_(*t*)/*U*_*c*_(*t*)〉_*V*_, the average of *V*_*c*_(*t*) and *U*_*c*_(*t*) across all streams and times such that *V*_*c*_(*t*) = *V*. That is, *M*(*V*) measures the average user posting activity at a given information load value *V*.

Chat patterns in a stream may potentially change in response to the fact that the chat is, or is not, happening during a broadcast, rather than a simple increase in overall activity. We want, therefore, to consider only data collected during broadcasts. This poses a challenge, as the explicit information about the starting and ending of broadcasts is not available in our data. Streamers typically stream only for a few hours a day, but the chat room is available without interruption. As a result, messaging activity in a stream exhibits surges, interspersed between long periods of little or no activity, as shown in figure 2. Since communication is more likely to occur during broadcasts, we filter out periods of inactivity, using a simple clustering heuristic (see *Methods* for more information about broadcast detection).

A second challenge has to do with the presence of messages generated by non-human accounts, or *bots*. It is common for streamers to automate the management of their stream. Indeed, our preliminary manual inspection revealed an abundance of bot-generated messages. It is unlikely that bots suffer from information overload, and they could skew the estimates of our metrics. At the same time, bot activity contributes to the information load of a stream as much as human activity does. Therefore, while we do not remove the contributions from bots in computing the number of messages, we remove bots from the user activity—the *y*-axis of figure 1*c*. To compute all other metrics we filter out bot-generated messages in a similar manner.

We detect bots in our dataset using a simple heuristic based on the compressibility *ρ* of the text of the messages (see *Methods* for more information about bot detection).

### 2.1. The case for overload and alternative scenarios

**Figure 3.**
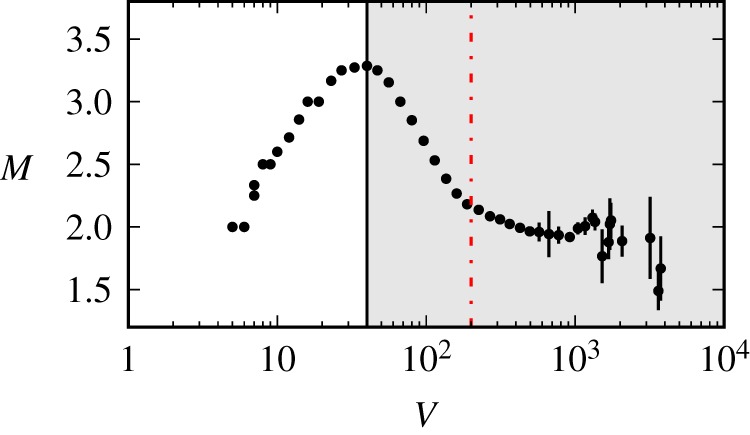
User activity (messages *per capita* *M*) as a function of information load (number of messages *V*). The grey shaded area indicates the regime of overload—for a comparison, cf. figure 1*c*. We include only streams with at least 1000 messages and at least 100 users observed during the full observation window overall (*N* = 43 969). To compute *M*, we average across chunks of messages sampled every Δ*t* = 5 min. To mitigate fluctuations due to undetected bots, we estimate the average using the median instead of the sample average, and consider only chunks with number of users *U* > 1. Error bars represent standard error of the mean.

Figure 3 shows *M*, the average number of messages posted by a user, as a function of the information load *V*. The plot shows an inverted *U*-shape: *M* initially increases, peaks at V* ≃ 40 messages every 5 min (about one message every 7.5 s) and then decreases. The decay is at first abrupt, then around *V* = 200 messages every 5 min (equivalent to one message every 0.67 s), more steady.

This observation is consistent with our model of overload of figure 1*c*. A number of alternative explanations may, however, be considered.

**Figure 4.**
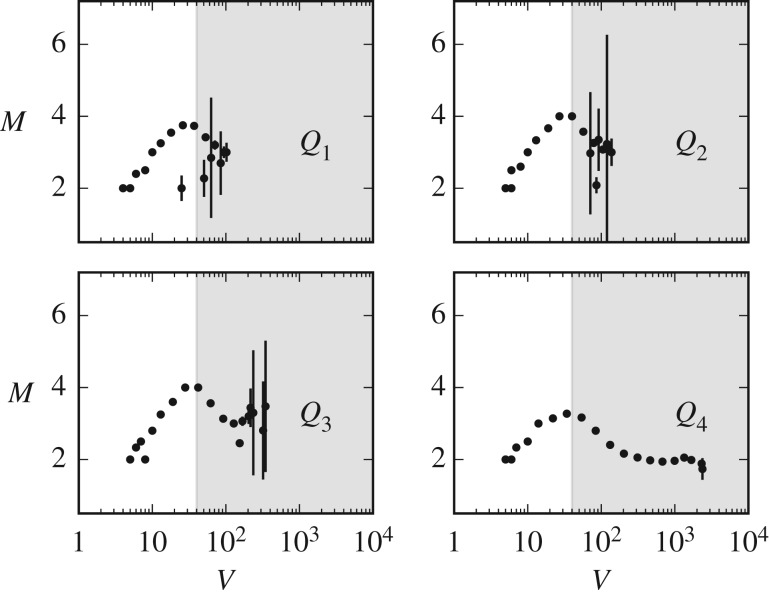
User activity (messages *per capita* *M*) as a function of information load (number of messages *V*), broken down for the quartiles of the distribution of users per streams (figure 8*b*). The group with least popular streams is denoted by *Q*_1_. To estimate *M*, similar steps as in figure 3 were used, with the exception that we only consider chunks with *U* > 2.

The first is that the above result could be a mere artefact of aggregating together streams with dramatically different activity levels. Streams with less users, and thus lower activity, could be responsible for the initial increase, while streams with more users, and thus higher activity, for the subsequent decrease. To rule out such an alternative explanation we group streams in four separate groups, each corresponding to a quartile of the stream popularity distribution of figure 8*b*, and repeat the analysis of figure 3 in each group. Despite increased fluctuations due to smaller sample sizes, figure 4 shows that the pattern still holds even when we restrict to streams with small or intermediate popularity levels.

The second alternative scenario is that the inverted *U*-shape is the by-product of grouping together individuals whose behaviour is actually independent of information overload. A famous example of this is the Simpson’s paradox [25], which is not rare in the realm of the social sciences [26]. In other words, we seek to quantify the extent to which an inverted *U*-shape relation between *V* and *M*, similar to that observed in figure 3, holds in our population of Twitch users when we consider users at the individual level.

To do so, we compute *M*_u_, the number of messages posted by each user (averaged across all time blocks with the same *V*) and we regress it against *V*. We fit a multi-level model to the data, which lets us account for variation among users. We use, in particular, a generalized linear model for count data, in which both slopes and intercepts may vary by individual. Specifically, we model the *i*th observation of the *j*th individual as:

2.1 $$M_{u}^{\left(i\right)}=\beta_{\;j[i]}+\alpha_{\;j[i]}V_{i},$$

where *α* and *β* both vary by individual *j*, and thus are indexed with the notation *j*[*i*] to denote that observations are grouped by individual. We fit two distinct models, respectively, for the sub- and supra-threshold regimes (V* = 40 messages every 5 min).

In the sub-threshold regime (*V* < V*), the intercept is 1.94 (s.e. 0.03) messages every 5 min, and the slope is 0.03 (s.e. 0.00). In the supra-threshold regime (*V* > V*), the intercept is 3.31 (s.e. 0.04) messages every 5 min, and the slope is −0.02 (s.e. 0.00). The signs of the slopes—positive in the sub-threshold regime and negative in the supra-threshold regime—are consistent with an inverted *U*-shape relation.

**Figure 5.**
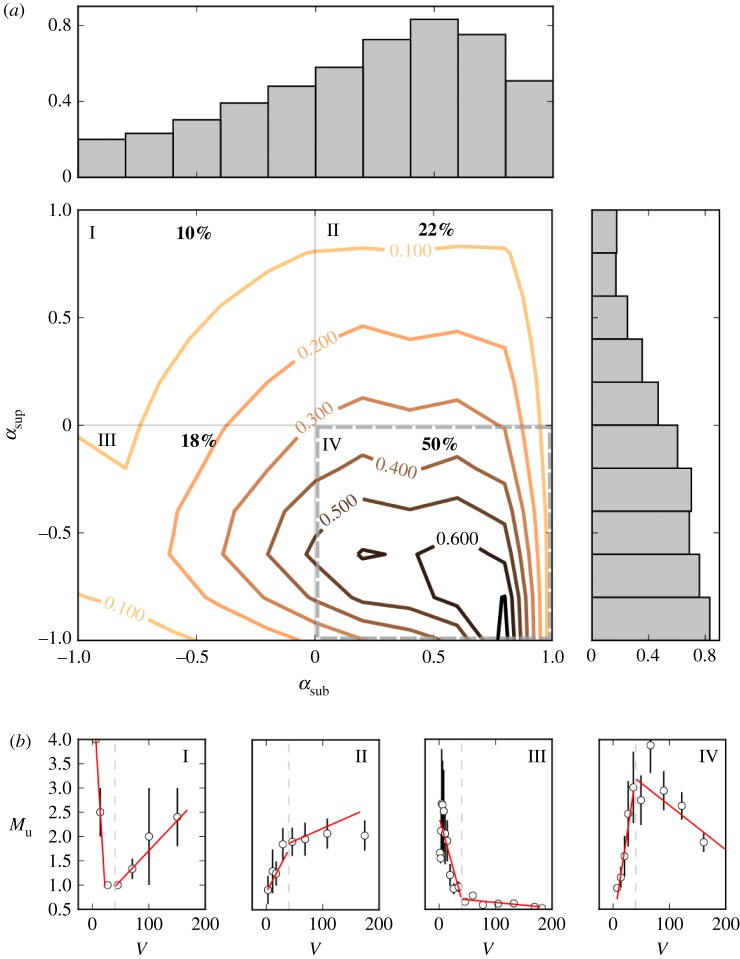
(*a*) The plot shows the distribution of a sample of users in the *α*_sub_ × *α*_sup_ space for V* = 40 messages every 5 min. Each point is obtained by regressing *M* on *V* in the sub- and supra-threshold regions, respectively. Users with at least 10 observations in either regions were selected. Points that fall within the S-E quadrant (highlighted) correspond to users whose behaviour is consistent with the inverted *U*-shape curve. (*b*) We selected one user at random from each of the four quadrants; in the lower panels (I–IV) we show their response curves and estimated *α*_sub_ and *α*_sup_ slopes (red lines). The dashed line corresponds to V*.

To get a better sense of the overall variation in the population, we also perform a visualization exercise in which, instead of the multi-level model described above, we fit a simple linear regression line to each user (and each regime). We compute slopes *α*_sub_ and *α*_sup_ of the two regression lines for both the sub- and supra-threshold regimes, and visualize their joint distribution. To compare values across different users, we standardize the data before the regression. We expect to find four groups of users based on the sign of the slopes of two regions. In figure 5, we show the distribution of users in the *α*_sub_ × *α*_sup_ space for V* = 40 messages every 5 min. The coefficient *α*_sup_ was estimated using data chunks whose value of *V* was in the range V* < *V* < 200 messages every 5 min, since for *V* > 200 estimates of *M* tend to be affected by too much statistical noise. In line with the multi-level fit, contour lines show that the majority (50%) of users have a behaviour consistent with the inverted *U*-shape curve model of information overload.

Finally, the last alternative scenario consistent with the observations in figure 3 is that the presence of events in the video stream may drive participation to the chat. A particularly virtuoso play, for example, may elicit praise in the chat.

Our data, lacking any video information, do not allow the direct detection of this kind of exogenous events. However, if patterns consistent with information overload were present also in the absence of a video broadcast, it would provide evidence that this alternative explanation cannot explain the observed patterns. Indeed, since broadcasts require the streamer to be online, there are plenty of periods during which the video of a stream is inactive. During these periods, the chat is still available, but the screen is either blank or set to a still image.^2^

**Figure 6.**
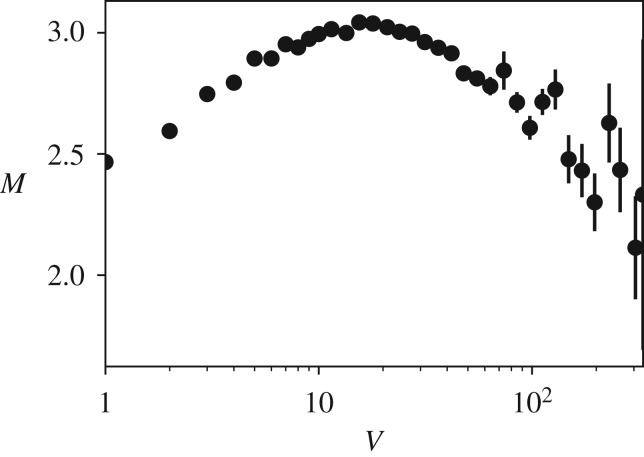
User activity (messages *per capita* *M*) as a function of information load (number of messages *V*) for periods of video inactivity (corresponding to unshaded areas in figure 2). During these periods the streamer is offline, and the video is either blank or set to a still image.

We repeated the analysis of figure 3, this time we used only data from inactive periods (the not-shaded areas in figure 2), and observe that our main finding—the inverted *U*-shape relation between information load and user activity—does holds, albeit in qualitative fashion (figure 6). Because the video feed is inactive, we can assume that the information load experienced by users is only coming from the chat, and thus *V* must be the driver of information overload.

### 2.2. Characterizing the conversational and overload regimes

We then characterize the content of messages produced in the two regimes. We use several structural and textual metrics to do so (see *Methods* for more information about textual and structural metrics).

**Figure 7.**
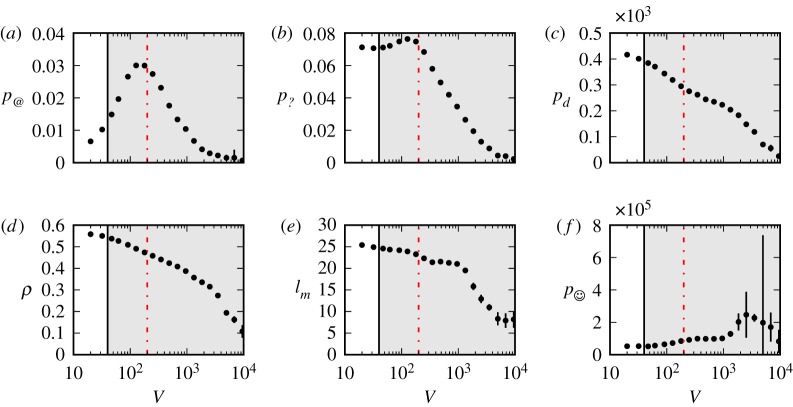
Information-based, lexical, and textual and structural metrics of information overload (see *Methods* for information about metrics). Quantities were estimated over chunks of messages collected every Δ*t* = 5 min. The same number of streams used for figure 3 was used. The grey shaded area corresponds to the same region in figure 3, while the red dot-dashed line corresponds to *V* = 200 messages. Error bars represent the standard error of the mean.

To identify the propensity to entertain conversations, we plot the frequency of mentions *p*_@_. In Twitch, like in Twitter, the ‘@’ symbol is used to mention other users. The number of mentions per time interval, as function of the number of messages *V*, is shown in figure 7*a*. The figure shows a qualitative behaviour similar to that of *M* and is amenable to similar interpretation. We control for alternative scenarios using the following strategy. The more popular a user is—for example—the more likely it is that they will be mentioned when they are not present in the chat or when, if present, they are not taking part to any conversation. Thus, counting individual @-mentions may lead us to assume a breakdown in conversation behaviour when no actual conversation had occurred in the first place. To control for any potential bias due to popular users we repeat the analysis, this time counting the number of mentioned individuals rather than @-mentions. We find no qualitative difference with the plot of figure 7*a*.

Question marks are another signal compatible with conversation or at least with the expectation to be noted, listened to, and possibly answered by the user population at large. The frequency of questions *p*_?_ (figure 7*b*) remains approximately constant for the whole conversation regime and part of the overload regime and drops dramatically around *V* = 200 messages every 5 min. Aside from peaking earlier than *p*_?_, the frequency of discourse markers *p*_*d*_ shows a qualitatively similar behaviour, decreasing steadily in both regimes (figure 7*c*).

The amount of information, expressed as a compression ratio *ρ* (see *Methods* section about metrics), is uniformly decreasing, indicating higher compressibility (figure 7*d*). This implies that, as the overall activity increases, the content becomes more repetitive, both within messages (e.g. emoticons repeated several times) and across messages, possibly due to increased use of copy-and-paste. In figure 7*e*, we plot the message length *l*_*m*_, and observe that messages get shorter as activity increases. When we estimate the frequency of emoticons and emotes *p*_☺_, we observe that, at the same time as messages become shorter, users resort to more emoticons and emotes (figure 7*f*). In the conversation regime, *p*_☺_ actually *decreases*, reaching its lowest approximately at the onset of the overload regime (as predicted by *M*).

### 2.3. Limitations

We acknowledge the following limitations in our methodology. The first has to do with text. Some of our textual features are based on English, like the list of discourse markers that we use to compute *p*_*d*_. Of course, there are plenty of non-English speakers on Twitch, and we do not filter out their messages in our data. Different discourse conventions from non-English speaking cultures may thus introduce bias in the results for *p*_*d*_, and perhaps even of *p*_?_. In part to mitigate for these assumptions, we apply content-agnostic features such as the compression ratio *ρ*. In the future, we plan to use language detection techniques to select only a subset of languages, and use language-specific lists of discourse markers.

The second is about the lack of metadata about the structure of the conversation. Because of the nature of the system, we cannot reconstruct actual conversation threads or reply sequences, as is possible for example, with Twitter data. We do mitigate for this by looking at markers of conversation, like the use of @-mentions, question marks and discourse markers. We also take care to exclude bots from our data when computing overload, and take into account other potential confounding factors.

Finally, it is worth mentioning that our dataset was collected in 2014; Twitch has undoubtedly changed a lot in the meantime. Because our main findings (e.g. figure 3) do not rely on any particular feature of the Twitch chat, we expect that analyses on more recent data would produce results consistent with ours. It is reasonable to expect that results related to bot detection and other features that strongly rely on the Twitch platform may be different from those we present here.

## 3. Discussion

The popularity of social media, combined with the pervasiveness of mobile devices, keeps an increasing number of people under a constant barrage of text, videos and audio inputs. This affects their ability to properly filter, comprehend, consume and ultimately act upon information. Such phenomenon is informally referred to as ‘information overload’. Prior work on information overload has highlighted effects at the individual level, and quantified the extent to which the ability to consume information is impacted by overload. The effect on the production of information taking place in a group setting has been devoted less attention. Leveraging Twitch chat conversations, we find strong evidence of two regimes of activity in online group interactions—a *conversation* and an *overload* regime (h1). The average number of messages *per capita* *M* follows an inverted *U*-shape relation with the total number of messages *V* that flow through the chat, suggesting an increase in participation during the conversation regime, and a decrease in the overload regime (h2). This is consistent with previous accounts of information overload focused on information consumption [15].

The transition between the two regimes is associated with strong, visible changes in the nature of the conversations (h3). The regime of low information load is marked by several characteristics that betray the presence of ongoing conversations: messages are long and varied; users interact with each other by means of questions and direct mentions; the usage of emoticons is kept to a minimum. The other phase is radically different: messages become shorter and more stereotypical, as evidenced by their higher text compressibility; conversation markers disappear, replaced by an increase of emoticons; users stop interacting with each other. Such a regime is more akin to a *cacophony*—a discordant mixture of distinct voices overlapping each other.

The transition (h4) between the two phases is gradual rather than abrupt. It is marked by two turning points. The first corresponds to the peak of user participation, around *V* = 40 messages every Δ*t* = 5 min. The second one, located around *V* = 200 messages per Δ*t*, is relative to changes in the textual structure of the messages. Around this point, the frequency of user mentions *p*_@_ peaks and the frequency of questions *p*_?_ starts dropping dramatically: the interactions become less and less one-to-one, or one-to-few.

These findings, which have been first revealed in the aggregate, considering averages over many conversations from many streams, are supported also at the individual level, and are not affected by Simpson’s paradox. We quantify the variation of behaviour by fitting a multi-level model, finding that per-user output *M*_u_ follows an inverted *U*-shape curve as a function of information rate *V*, similarly to what happens for corresponding average quantity *M*; see figure 3. We rule out several explanations competing with the *overload effect*. Our observations cannot be explained in terms of heterogeneous stream popularity—similar patterns are visible also when we break down the data by stream popularity (figure 4)—nor by exogenous increases of *U*—for example, due to a particularly good or entertaining move in the game—because any increase in *U* must correspond to an increase in *V*, due to the fact that our data do not include non-chatting viewers. Finally, they cannot be due to salient events in the video itself, since they hold also when the video stream is inactive (figure 6).

Beside offering an unprecedented source of data for measuring overload at very high information loads, it should also be noted that Twitch conversations could be an important subject of investigation in their own right. The Twitch chat is indeed a powerful community-building tool [20], and by engaging with their viewers through the chat, streamers build rapport with their audience, especially when the scale of the audience is not too large. Knowing when a conversation may tip into overload could thus be a useful parameter for platform operators and streamers alike.

More generally, Twitch chat conversations offer a useful environment for understanding the limits of information processing in interpersonal communication. The present findings could find application in other contexts, such as social media feeds, collaborative environments and intelligent systems with humans in the loop.

In conclusion, we studied the dynamics of Twitch chat conversations. To our knowledge, this is the first time a large systematic sample of logs from the Twitch chat has been analysed. We provide quantitative measurements for the onset of information overload at both the collective and individual level, and describe its effects on the overall structure and dynamic of the group. Our finding may inform designers of social media user interfaces (UIs). For example, it could be beneficial to introduce an automatic detector of possible overload based on the rate of messages, or visual aids for users to cope with overload.

## 4. Methods

### 4.1. Twitch

Twitch started in 2007 as a ‘social TV’ experiment under the name of *Justin.tv*, giving users the capacity to broadcast their own video streams. With the rise of popularity of electronic sports, or *eSports*, it has rapidly become one of the most popular live-streaming platforms on the Internet [27,28]. The majority of streams on Twitch are about video games, played by both amateur and professional players [20]. According to in-house statistics, Twitch rivals with traditional television networks (e.g. CNN) in terms of viewership, with 100 million monthly viewers in 2015. It also accounts for 1.8% of the overall peak-time Internet traffic, ranking behind only to Netflix, Google and Apple [29].

### 4.2. Data

Our data include all messages posted to any public Twitch stream in the period 26 August–10 November 2014 (76 days). We counted a total number of 1 275 396 751 messages, posted in 927 247 streams (average: 1375 messages per stream) by 6 716 014 users (average: 190 messages per user). Among all streams, 532 094 were active for at least 2 days, 319 451 had only one active user, and 166 870 had more than 100 messages. Among all users, 4 930 052 were active for at least 2 days, 5 015 079 participated in at least two streams, and 1 032 766 posted at least 100 messages.

The dataset was collected as a dump of the chat logs of all Twitch streams, and included only minimal metadata. We have very little structured information; it is impossible, for example, to track actual conversation threads without any additional pre-processing. A user (resp. stream) is logged in our dump only if they sent (received) a message. Therefore, users (or streams) who did not post anything during the observation window are not included in our data dump. The above figures, therefore, may not reflect the total activity of all users—viewers or streamers—on Twitch.

**Figure 8.**
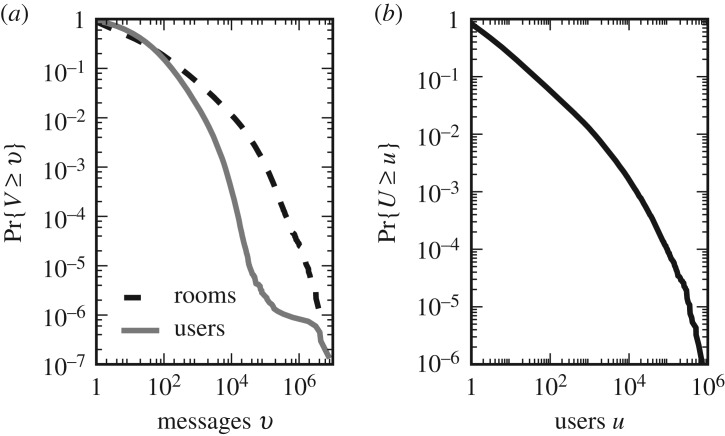
(*a*) Distribution of the number of messages per stream (black dashed line) and per user (solid grey line). (*b*) Distribution of the number of users per stream (stream popularity). All quantities show broad distributions.

In figure 8, we plot the distributions of messages and users. The number of messages produced in a stream (figure 8*a*) spans several orders of magnitude, with a median of seven messages. The same happens for the number of users posting in a stream (figure 8*b*), a rough proxy of its popularity, and for the message count of each user (figure 8*a*). Some users have written more than one million messages, while most only a few.

### 4.3. Broadcast detection

Let us consider a stream *c*. To detect broadcast periods, we sample the volume of messages *V*_*c*_(*t*), *t* = *n*Δ*t*, *n* = 0, 1, 2, … , at intervals of Δ*t* = 5 min. We then consider the time average of the message volume $\bar{V}={\left\langle V_{c}(t)\right\rangle }_{t}$ and define a symbolic sequence *S*_*c*_(*t*) where:

$$S_{c}(t)=\left\{ \begin{array}{c} A\;\;\text{if }V_{c}(t)\geq \bar{V},\;\\ I\;\;\text{if }V_{c}(t)<\bar{V}.\; \end{array}\right.$$

Examining *S*_*c*_(*t*), we noticed that sub-sequences AIA and IAI, i.e. below- or above-average spikes shorter than Δ*t*, would sometimes occur within longer sequences of As or Is, respectively. We replaced these fluctuations with AAA, and III respectively, assuming that a broadcast would not stop for such a very brief period. Finally, we defined the sequence IIA as the beginning of a broadcast, and AAI as the ending, and recorded the respective timestamps. If two consecutive broadcasts were separated by less than 60 min, we merged them together, assuming that consecutive broadcasts separated by such a short period would be highly unlikely. Figure 2 shows, as grey shaded areas, the detected broadcast periods for the example stream in our data.

### 4.4. Bot detection

Automated accounts, or *bots*, are known to be present in live-streaming services like Twitch. There are many types of bots operating on Twitch. Some report the status of the game or ranking of players, some post advertisements on behalf of the streamers, and some greet those who log into the stream. Some bots generate messages in several streams, while others are active only in one. Bots can be used either for abuse or for legitimate purposes. Abuse include spamming, trolling, or inflation of viewing statistics of videos (view bot). Since view bots do not normally post messages, our results cannot be affected by them, but could be still affected by the presence of bots that do post messages. Bots are known to produce messages at a rate higher than what is ‘normal’ for a human; and it is reasonable to expect that these messages will be also more repetitive. Based on this intuition, to detect bots in the chat log data we adopted two revealing features: the average inter-message time *τ*, and the compression ratio *ρ*. The compression ratio is defined as $\rho =\hat{S}/S$, where *S* is the length (in bytes) of the string obtained by concatenating all messages by the same user, and $\hat{S}$ is the length (in bytes) of the string obtained by compressing *S* with an off-the-shelf compression algorithm.^3^ The compression ratio estimates the information content of the messages of a user. In using this approach, we are motivated by the notion of Kolmogorov complexity [31].

When estimating *τ*, we need to take into account the fact that neither bots nor humans are active at all times. We, therefore, compute the average inter-message time only during ‘active’ periods: a user is considered in an active period if she has produced at least one message in the previous hour. If not, the inter-time is discarded and a new active session begins with the next message. The average *τ* is then estimated across all sessions with at least two messages.

We then considered all users who had been active more than one day and produced at least 10 messages (865 551 users). Note that we restrict to this subset of users for the one and only purpose described in this paragraph; all the other results of the manuscript are obtained on the full sample of users. We used a stratified sampling approach on *τ* and *ρ* to randomly selected 256 users from this population. Finally, we manually inspected all their messages, and labelled them with one of the following categories: *bot*, *human*, *copy-paster*, *non-English*, and *ambiguous*. The ‘copy-paster’ label is meant to capture users whose complete production consists only of one or more brief, fast sequences composed by the same, copy-pasted, message. Out of 256 users, we identify 49 bots, 92 humans and 59 copy-pasters. The remaining users were either ‘ambiguous’ (i.e. we could not determine whether it was a human or a bot) or ‘non-English’. We discarded these latter two groups from the bot detection analysis of this section.

**Figure 9.**
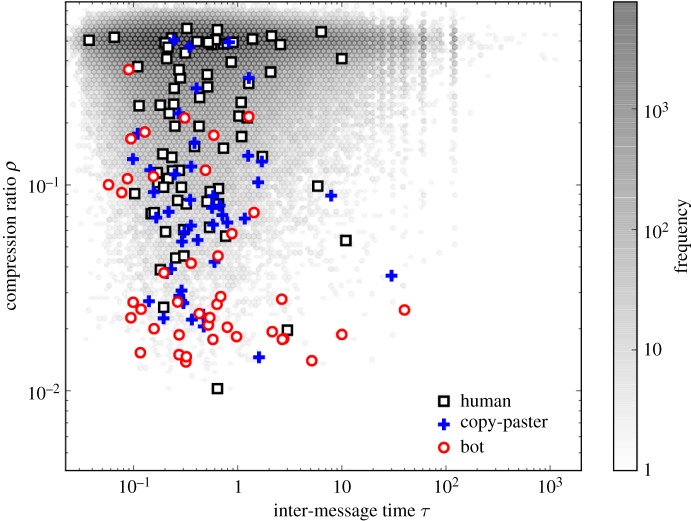
Bot classification. Manually labelled examples for the three classes ‘bot’, ‘human’ and ‘copy-paster’ are shown in the *τ* × *ρ* feature space. As a reference, the population density of users from which these examples were drawn is shown, with shades of grey mapped into the frequency of users in each bin.

Figure 9 shows the distribution of labelled examples in the (*τ*, *ρ*) plane and, as a reference, the general population distribution used for sampling. It suggests that, while *ρ* offers discriminating power, surprisingly, *τ* does not.

Conservatively, we retained only users with *ρ* ≥ 0.44, which corresponds to the maximum value of *ρ* observed for a bot among our 256 user sample. The total number of users removed turned out to be only 43 026 (0.5% of the reference population). The drastic filtering criterion adopted here is simple, but removes the great majority of bots at a relatively low price in terms of population reduction. This is important, since lacking a hold-out set, we cannot evaluate the precision of the method, and thus any potential impact due to classification mistakes should be mitigated by the small amount of users removed in this way.

### 4.5. Texual and structural metrics

In addition to *M*, to characterize the shift from the conversation to the overload regime, we compute the following textual and lexical features: the message length *l*_*m*_; the frequency of questions *p*_?_, measured by counting messages ending with a question mark sign; the frequency *p*_@_ with which users address each other with an @-mention; *p*_*d*_, the relative frequency of discourse markers, i.e. colloquial expressions such as ‘oh’, ‘well’ or ‘of course’ [32] (see electronic supplementary materials for the list of discourse markers); the fraction of emoticons and emotes *p*_☺_; and the average block compression ratio *ρ*. The quantities above have been chosen to help reveal the presence of conversations (*p*_@_, *p*_?_, *p*_*d*_), or a state of overload (the necessity of succinctness, *l*_*m*_, the (lack of) information exchanged *ρ*, the excessive use of emoticons *p*_☺_).

Frequencies *p*_?_ and *p*_@_ were computed at the level of messages, while *p*_*d*_ was computed at the level of words, breaking tokens in correspondence of white spaces, after transforming all text to lower case.

To compute *p*_☺_, we used the following approach. In Twitch, beside standard emoticons such as ‘:-)’ and ‘:-(’, it is also customary to use *emotes*—short text codes associated with small images that are rendered automatically in-line within the text. The Twitch software recognizes a list of approximately 190 standard emotes. Moreover, streamers can define additional emotes for their stream, which are available to viewers who pay a small monthly subscription fee.

We collected both kinds of emotes from the most comprehensive online resource that we could find.^4^ We found 16 763 subscription emotes, with no guarantee to have exhausted their list. To compute the probability of occurrence of these emoticons/emotes in a message, we first need to parse the text of the messages for their occurence. To do so, we break messages into short sub-strings of varying size called *k*-shingles [33]. We opted for shingling over a more common word tokenization strategy because emotes are often copied and pasted in sequence without separating white spaces. The maximum length of emotes in our list is 24, thus, we varied the value of *k* accordingly. For each value *k* we created a bag of shingles. We then merged all the bags, obtaining a total of *N* distinct shingles. Finally, we defined *p*_☺_=*N*_☺_/*N*, where *N*_☺_ was the number of shingles that matched any of the emotes in our list.

Similarly to what was done for the bot detection step, we quantify the information content of each message block with compression. For each stream and 5 min interval (chunk), we first collect and concatenate all the messages occurring in the chunk, and then compute the compression ratio for the concatenation. We finally compute *ρ*(*V*) as the average ratio across chunks with the same information load *V*.

## Supplementary Material

List of Discourse Markers

Reviewer comments

## Footnotes

1 On Twitch each user has a stream, but the majority of streams do not feature any content, and presumably are not watched by anybody. Since we have only access to the logs of chat messages, we do not have any data about these streams.

2 At the time of the collection of our data, if the streamer was offline the video would just show a blank screen or a still image with information about the next scheduled broadcast. Nowadays, users can also choose to play a video on demand of a past broadcast. In that case, the chat of the event would be replayed accordingly. At the time our data were collected, neither feature was available on Twitch, so people would simply chat without a video stream.

3 To perform the compression, we use the deflate algorithm, as implemented in the zlib library v.1.2.6 [30].

4 The data of the Twitch emotes are available at: https://twitchemotes.com/.

## Data accessibility

A subset of the chat logs dump from Twitch used in this work is provided, to help replicate the central findings of this work (https://doi.org/10.5281/zenodo.1182793). Data are aggregated and include the number of messages posted in each channel and the number of users posting them, sampled at intervals of 5 min. To protect the identity of the users in this data collection, message contents and user names are not included in this dataset. Stream names have been replaced with numeric IDs. No additional filtering or data cleaning operation has been applied to this data. Replication code is publicly available (https://doi.org/10.5281/zenodo.3369263).
