# Supplementary material for: Information overload in group communication: from conversation to cacophony in the Twitch chat
Source: R Soc Open Sci. 2019 Oct 9;6(10):191412. doi: 10.1098/rsos.191412 (PMC6837236; doi:10.1098/rsos.191412)
Supplement: Reviewer comments [file rsos191412_review_history.pdf]

THE ROYAL SOCIETY
PUBLISHING

ROYAL SOCIETY
OPEN SCIENCE

Information overload in group communication: from conversation to cacophony in the Twitch chat

Azadeh Nematzadeh, Giovanni Luca Ciampaglia, Yong-Yeol Ahn and Alessandro Flammini

Article citation details
R. Soc. open sci. **6**: 191412.
http://dx.doi.org/10.1098/rsos.191412

Review timeline

Original submission:	15 August 2019
Final acceptance:	8 September 2019

Note: Reports are unedited and appear as submitted by the referee. The review history appears in chronological order.

Note: This manuscript was transferred from another Royal Society journal with peer review.

Review History

Decision letter (RSOS-191412.R0)

08-Sep-2019

Dear Dr Ciampaglia:

It is a pleasure to accept your manuscript entitled "Information Overload in Group Communication: From Conversation to Cacophony in the Twitch Chat" in its current form for publication in Royal Society Open Science.

On reviewing the changes you have implemented in response to earlier review, the Editors are satisfied that your paper may be accepted.

You can expect to receive a proof of your article in the near future. Please contact the editorial office (openscience_proofs@royalsociety.org and openscience@royalsociety.org) to let us know if you are likely to be away from e-mail contact -- if you are going to be away, please nominate a co-author (if available) to manage the proofing process, and ensure they are copied into your email to the journal.

Reports © 2019 The Reviewers; Decision Letters © 2019 The Reviewers and Editors; Responses © 2019 The Reviewers, Editors and Authors. Published by the Royal Society under the terms of the Creative Commons Attribution License http://creativecommons.org/licenses/by/4.0/, which permits unrestricted use, provided the original author and source are credited

Due to rapid publication and an extremely tight schedule, if comments are not received, your paper may experience a delay in publication.

Thank you for your fine contribution. On behalf of the Editors of Royal Society Open Science, we look forward to your continued contributions to the Journal.

Kind regards,
Andrew Dunn
Royal Society Open Science Editorial Office
Royal Society Open Science
openscience@royalsociety.org

on behalf of Dr Cecilia Mascolo (Associate Editor) and Professor Marta Kwiatkowska (Subject Editor).

Follow Royal Society Publishing on Twitter: @RSocPublishing
Follow Royal Society Publishing on Facebook:
https://www.facebook.com/RoyalSocietyPublishing.FanPage/
Read Royal Society Publishing's blog: https://blogs.royalsociety.org/publishing/
